# Molecular characterization and phylogenetic analysis of orf virus isolated from goats in Sokoto metropolis, Nigeria

**DOI:** 10.2144/fsoa-2020-0162

**Published:** 2021-04-20

**Authors:** Nafi'u Lawal, Mubarak Ibrahim, Dauda Ayomide Onawala, Muhammad Bashir Bello, Rabiu Muhammad Aliyu, Yusha'u Shu'aibu Baraya, Abdullahi Aliyu, Aliyu Musawa Ibrahim, Aliyu Sa'adu

**Affiliations:** 1 Department of Veterinary Microbiology, Faculty of Veterinary Medicine, Usmanu Danfodiyo University, Sokoto, Nigeria; 2 Central Veterinary Research Laboratory, Faculty of Veterinary Medicine, Usmanu Danfodiyo University, Sokoto, Nigeria; 3 Center for Advance Medical Research and Training, Usmanu Danfodiyo University, Sokoto, Nigeria; 4 Department of Veterinary Pathology, Faculty of Veterinary Medicine, Usmanu Danfodiyo University, Sokoto, Nigeria; 5 Department of Veterinary Public Health & Preventive Medicine, Faculty of Veterinary Medicine, Usmanu Danfodiyo University, Sokoto, Nigeria; 6 Department of Animal Health & Production Technology, College of Agriculture & Animal Science, Wurno, Sokoto, Nigeria

**Keywords:** *B2L* gene, contagious ecthyma, goat, orf virus, phylogenetic analysis

## Abstract

**Aim:**

The aim of this study was to molecularly characterize orf virus isolated from clinical infections in goats in Sokoto metropolis.

**Materials & methods:**

Embryonated chicken eggs were used to isolate orf virus according to the established protocol. Viral DNA was extracted and full coding region of *B2L* gene was amplified by polymerase chain reaction, sequenced and blasted for identification and phylogenetically analyzed.

**Results and discussion:**

The *B2L* gene sequences of the isolate showed slight variability (96–98.7%) with the reference sequences as it clustered within the same clade with Korean, Zambian and Ethiopian strains, signifying a close genetic relationship. Unique amino acid substitutions were noted. This is the first genetic characterization of *B2L* gene of orf virus circulating in Nigeria.

**Conclusion:**

This study has provided in sight into the genetic diversity of orf virus in the study area.

## Lay abstract

Contagious ecthyma has for long been affecting the goat and sheep population in Nigeria leading to huge economic losses to livestock farmers through death and poor market value of the affected animals. Recently, increased death due to this disease has been reported in north-central Nigeria, but the nature of the disease-causing agent has not been reported yet. It is the aim of this study to understand the nature of the virus causing the disease in goats in Sokoto using molecular biology techniques so as to facilitate better vaccine design for the effective control of the disease in Nigeria.

## Background

Contagious ecthyma (CE) is a highly contagious viral disease of small ruminants such as sheep and goats [1] that occasionally affects camels [2] and wild ruminants with huge economic impact on the livestock industry [2–6]. Clinically, the disease is associated with skin lesions such as erythema, macules, papules, vesicles, pustules and crusts on the lips, tongue, teat, nose, hooves and other parts of the body [6] especially in young lambs and kids [3,7]. The disease is generally selflimiting, however, secondary bacterial infection may complicate the situation, causing inappetence, severe emaciation and death of the affected animals [1]. Epidemiological evidence indicated morbidity of 60% but the mortality is usually low unless complicated by secondary bacterial infection [8] where it can reach up to 10 and 93% in kids and lambs respectively [9] and even 100% in adult goats [4]. The disease is also of zoonotic significance, causing ulcerative lesions or nodules on the hands of high risk individuals such as veterinarians, butchers and other animal handlers [10]. Except in immunocompromised patients, most human cases of contagious ecthyma are localized and heal spontaneously [11].

The aetiology of CE is orf virus, a member of the genus *Parapoxvirus* in the family *Poxviridae* [12]. The genetic material of the virus is a linear dsDNA [13] of 134–139 kb in size [3,13]. It exhibits high GC content of about 66% [14] and is generally organized into conserved central portion and variable terminal regions [15]. The central portion has a number of genes including the *B2L* gene, that encodes the immunogenic major envelope protein p42K [3,4,16,17]. This gene has been extensively used for molecular detection and diagnosis [18] as well as phylogenic analyses of various orf virus isolates [3,13,19].

Laboratory diagnosis of CE can be achieved using electron microscopy, histopathology and serological tests such as fluorescent antibody technique, virus neutralization test, agar gel immunodiffusion and ELISA [1,3]. Nowadays, confirmation of CE is achieved using polymerase chain reaction (PCR) which has been shown to be highly specific and sensitive [1,13,16,19]. Using PCR, sequencing and phylogenetic analysis, genetic characteristic and diversity of orf virus has been described in many countries around the world including China [5,19,20], Taiwan [13], Malaysia [7], India [12], Uruguay [3] and a few African countries such as Tanzania [21], Ethiopia [17,22], Egypt [23,24], Gabon [25] and Sudan [26,27]. To date, outbreaks of CE in Nigeria are largely reported based on clinical manifestation of the disease and PCR to confirm cases [1,2,4] but no literature on the molecular characterization of the circulating orf virus isolates is currently available in Nigeria as at the time of this study. Therefore, in the present study we reported for the first time the isolation, molecular detection and phylogenetic characterization of orf virus obtained from a flock of goats in Sokoto metropolis, north-west Nigeria.

## Materials & methods

### Sample collection, transport & processing

**Figure 1.**
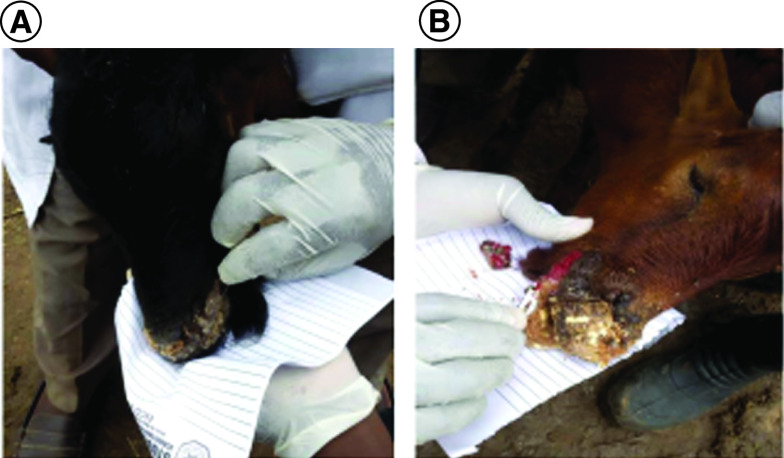
Sample collection from goats with suspected orf virus infection. **(A)** Sample 1 from male RSG-WAD cross. **(B)** Sample 2 from female RSG.

Suspected outbreak of CE was reported in a goat farm located in More area, Sokoto metropolis (13.0059° N, 5.2476° E) in May 2019. On visitation to the farm, a flock of 30 goats consisting of Red Sokoto goat (RSG) and crosses of RSG with West African Dwarf (WAD) was observed (eight males, 22 females). Five of the males were less than a year old, while three were 2–3 years of age. The females on the other hand consisted of ten adults (2–4 years old) and 12 kids (less than a year old). Physical examination of the two affected goats (2-year old male RSG-WAD cross and 3-year old female RSG) revealed scab lesions on the ears, lips and nose (Figure 1). Two samples (1 and 2) involving thick brown scabs were scrapped on clean paper from the two affected goats and immediately transferred in a sterile sample container containing PBS (pH: 7.2–7.4), placed on ice and immediately transported to the Central Veterinary Research Laboratory (Usmanu Danfodiyo University, Sokoto) for analysis. After sample collection, the two animals were isolated from the rest of the flock and were treated with long acting oxytetracycline 20% at 1 ml/20 kg body weight to prevent secondary bacterial infection and the scraped lesions were scrubbed with povidone iodine and sprayed with gentian violet to facilitate wound healing.

The samples were homogenized in PBS using tissue homogenizer and centrifuged at 1000 × g for 15 min to harvest the supernatants for storage at -20°C until needed for further analysis.

### Virus isolation

Nine–11 days old specific antibody free chicken embryonated eggs (CEE) were purchased from the Vaccine Research Division of the National Veterinary Research Institute (Vom, Nigeria). The eggs were candled to ensure their viability and were later inoculated with 500 μl each of the two prepared samples via the chorioallantoic membrane (CAM) route as described by [28], sealed and labeled appropriately. The eggs were incubated and observed daily for 5–7 days during which eggs with dead embryos were chilled at 4°C. At the end of the incubation period, those embryos still alive were placed at 4°C overnight. Subsequently, the CAM was harvested and observed for the development of pock lesions before being stored at -20°C until processed.

### DNA extraction

Infected CAM harvested from the eggs inoculated with the two samples were used for genomic DNA extraction using DNA Mini kit (QIAGEN, Hilden, Germany). Briefly, about 250 mg of CAM was homogenized and placed in a 1.5 ml micro centrifuge tube. Lysis buffer and proteinase K were added followed by incubation at 56° C in a water bath until complete lysis of the tissues occurs. DNA was then extracted according to the manufacturer’s instructions, eluted with 50 μl elution buffer and stored at -20° C.

### Polymerase chain reaction

The ORFVB2LF1 5′-TCCCTGAAGCCCTATTATTTTTGTG-3′ and ORFVB2LR1 5′-GCTTGCGGGCGTTCGGACCTTC-3′ specific forward and reverse primers described by Hosamani *et al.* [29] were used to amplify the complete *B2L* gene of the orf virus (ORFV) with the aid of Toptaq PCR mastermix (QIAGEN) according to the manufacturer’s instructions. The mixture was briefly centrifuged and placed in the thermocycler. Amplification was carried out using initial denaturation at 94°C for 3 min, 35 cycles of 94°C for 30 sec, 60°C for 30 sec and 72°C for 1 min. Final extension was performed at 72°C for 5 min. The amplified products were then analyzed by electrophoresis on a 1.5% agarose gel containing 0.5 ng/ml ethidium bromide in Tris-acetate-EDTA (TAE) buffer according to Lawal *et al.* [30]. The amplicons were viewed using a GelDoc imaging system (BioRad, CA, USA).

### DNA sequencing, phylogenetic & evolutionary analyses

PCR positive samples were sent to (Inqaba Biotechnical Industries [Pty] Ltd, Pretoria, South Africa) for Sanger sequencing. The sequencing company reported that only one of the two samples passed quality control (QC) for downstream sequencing. Since the two samples were obtained from the same outbreak in the same flock, we asked the company to go ahead and sequence the sample that passed QC as the isolates in the two samples are highly likely to be the same. On receiving the result for the single sample, the Sequence was trimmed and subjected to BLAST similarity search using the BLASTN algorithm of the NCBI database, to confirm the identity of the virus. Subsequently, the obtained sequence was deposited in the GeneBank database with accession no MT272780 (available at https://www.ncbi.nlm.nih.gov/nuccore/MT272780). Reference sequences were downloaded and aligned with the sequence obtained in this study using ClustalW in the MEGA7 software [31]. Phylogenetic tree was constructed using the neighbor-joining method with 2000 bootstrap replicates using MEGA7 [31]. Evolutionary distances were inferred using Tamura–Nei model based on pair-wise sequence comparison with the use of MEGA7 software [31] between the isolates obtained in this study and the reference sequences.

## Results

### Virus isolation

Following the inoculation of the processed two scab materials into the specific antibody free CEE, pathologic changes in form of small grayish white foci (pock lesions) were observed on the harvested CAM membranes. These changes were not observed in the mock inoculated eggs. This signifies the successful presumptive isolation and identification of the virus.

### PCR & sequence analysis

**Figure 2.**
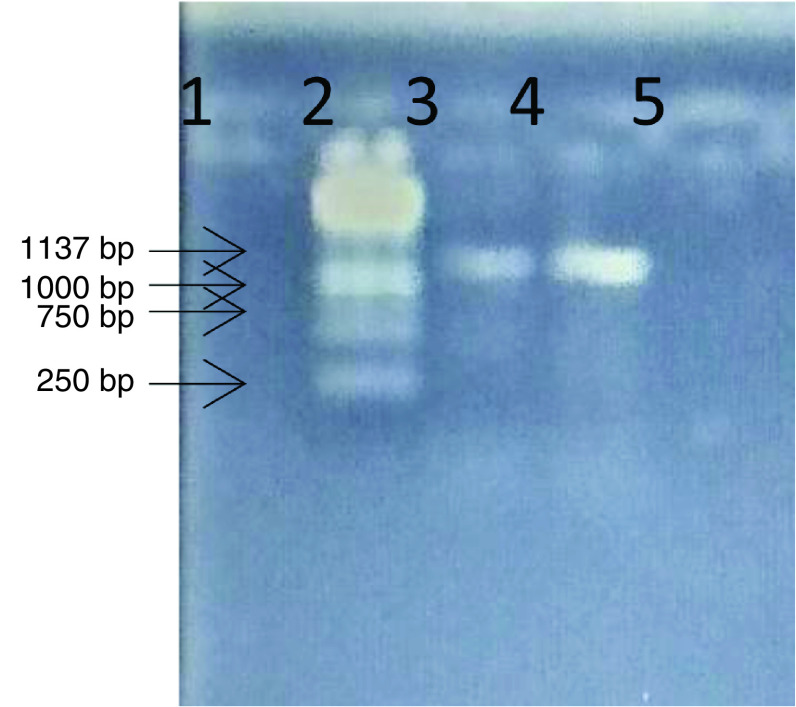
The PCR product showing the 1137bp fragments from sample 1 (well 3) and sample 2 (well 4, More_strain) with the 1kb DNA ladder (well 2) and the negative control (well 5). bp: Base pairs.

The PCR amplification of the CAM homogenate from the two samples yielded a product of the *B2L* gene fragment at the expected band size of 1137 bp when analyzed by gel electrophoresis (Figure 2). The PCR product of sample number 2 from the RSG (well 4) had higher band intensity compared with sample number 1 from the RSG-WAD cross (well 3). The negative control used showed no positive amplification.

**Figure 3.**
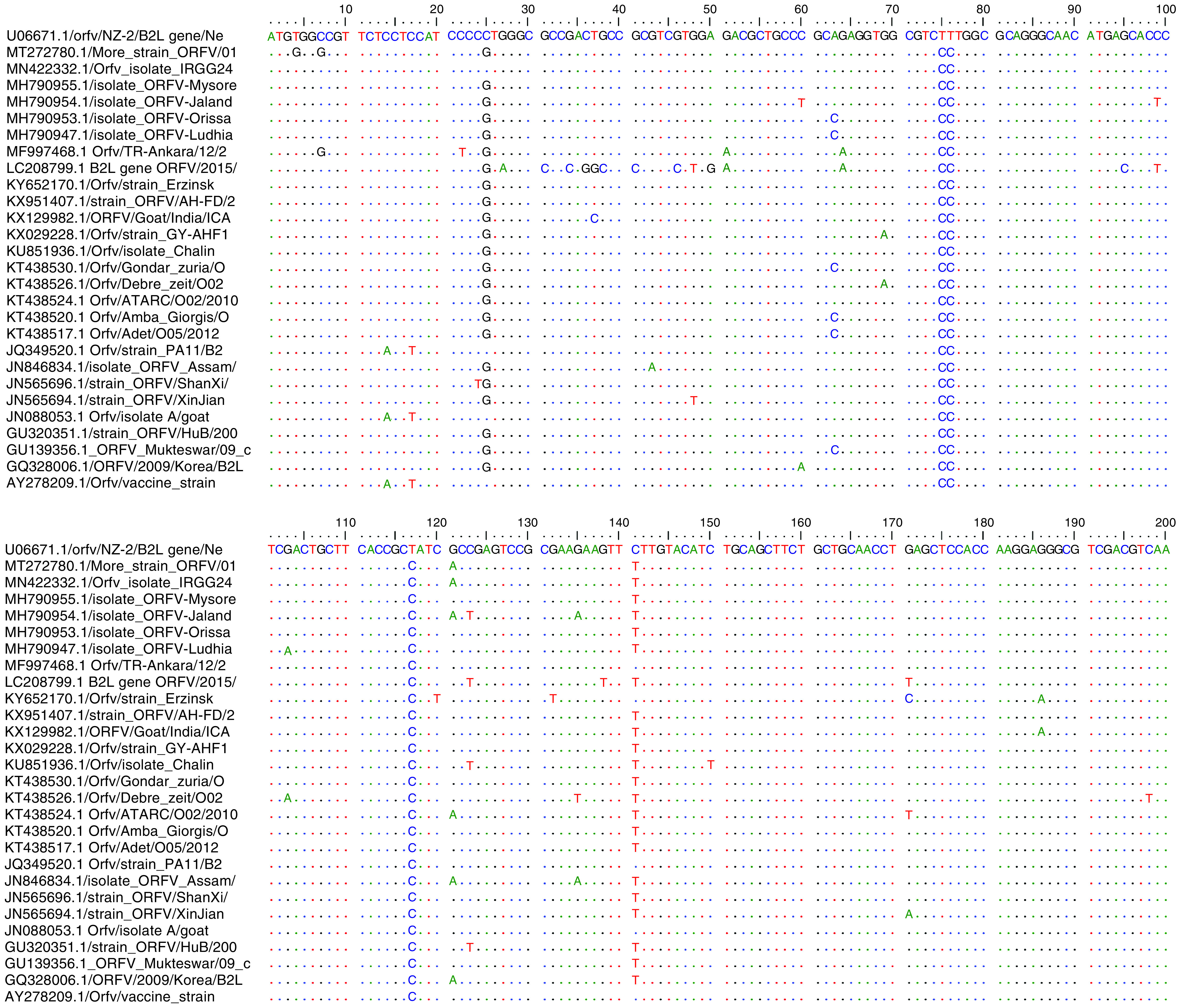

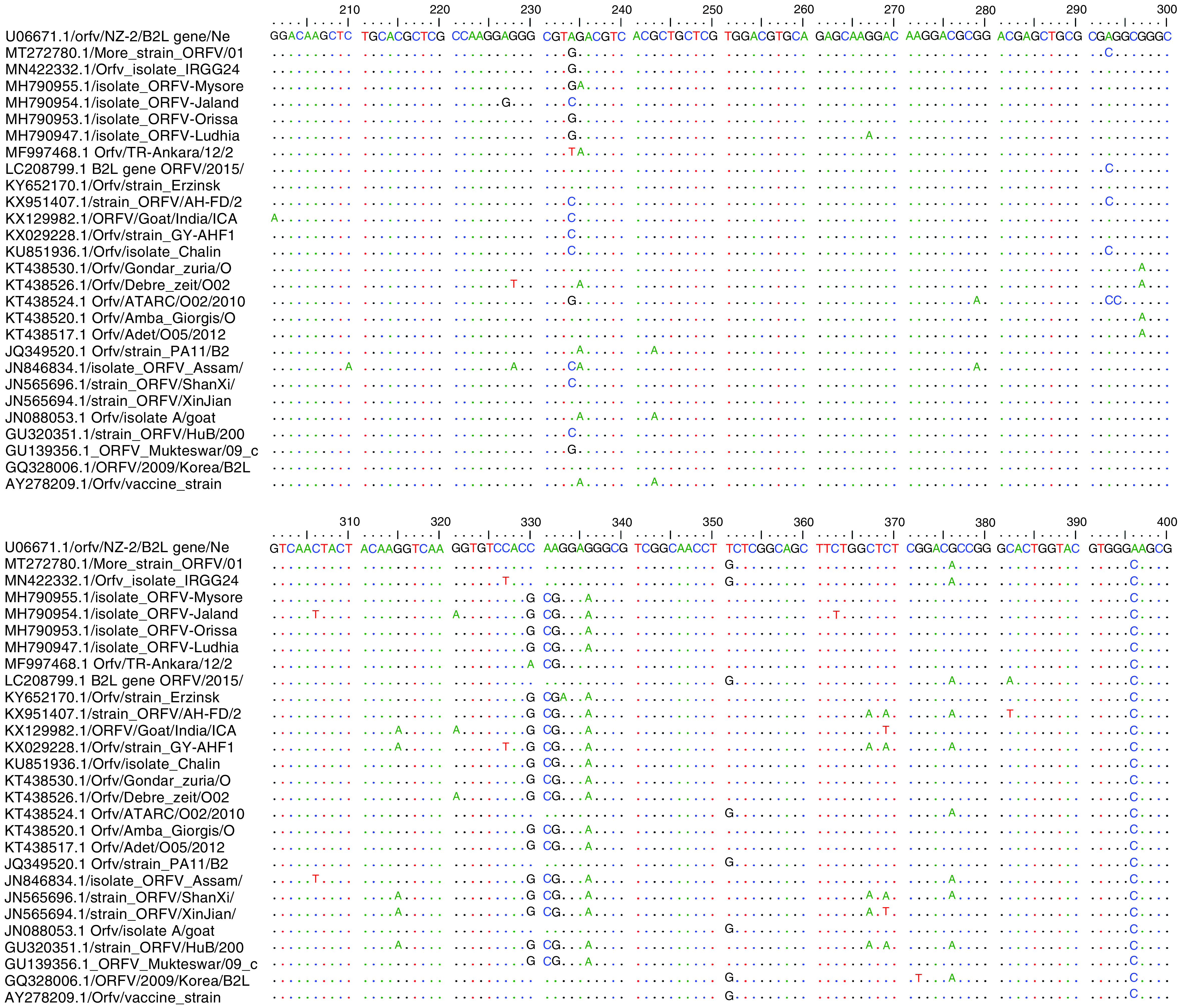

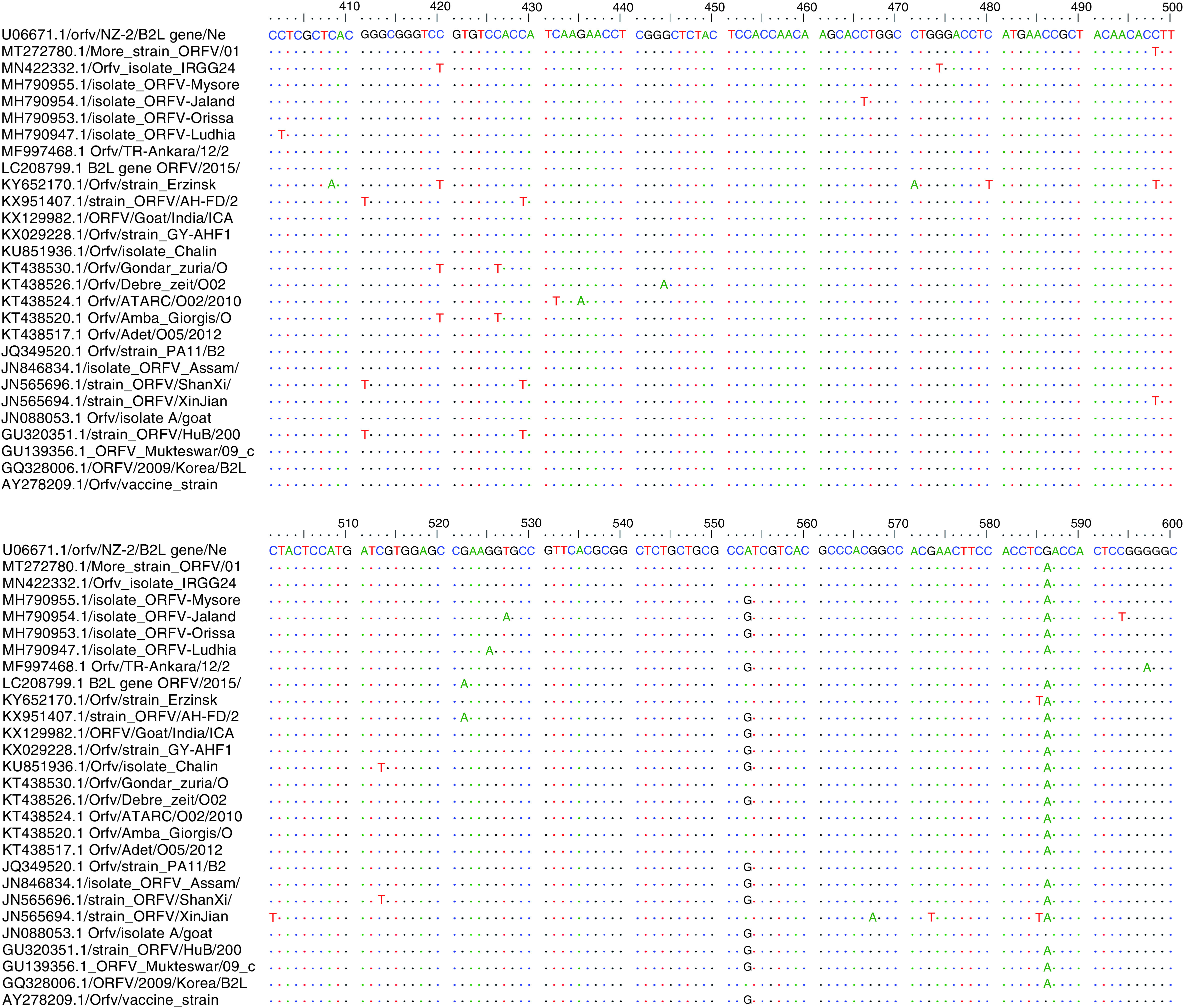

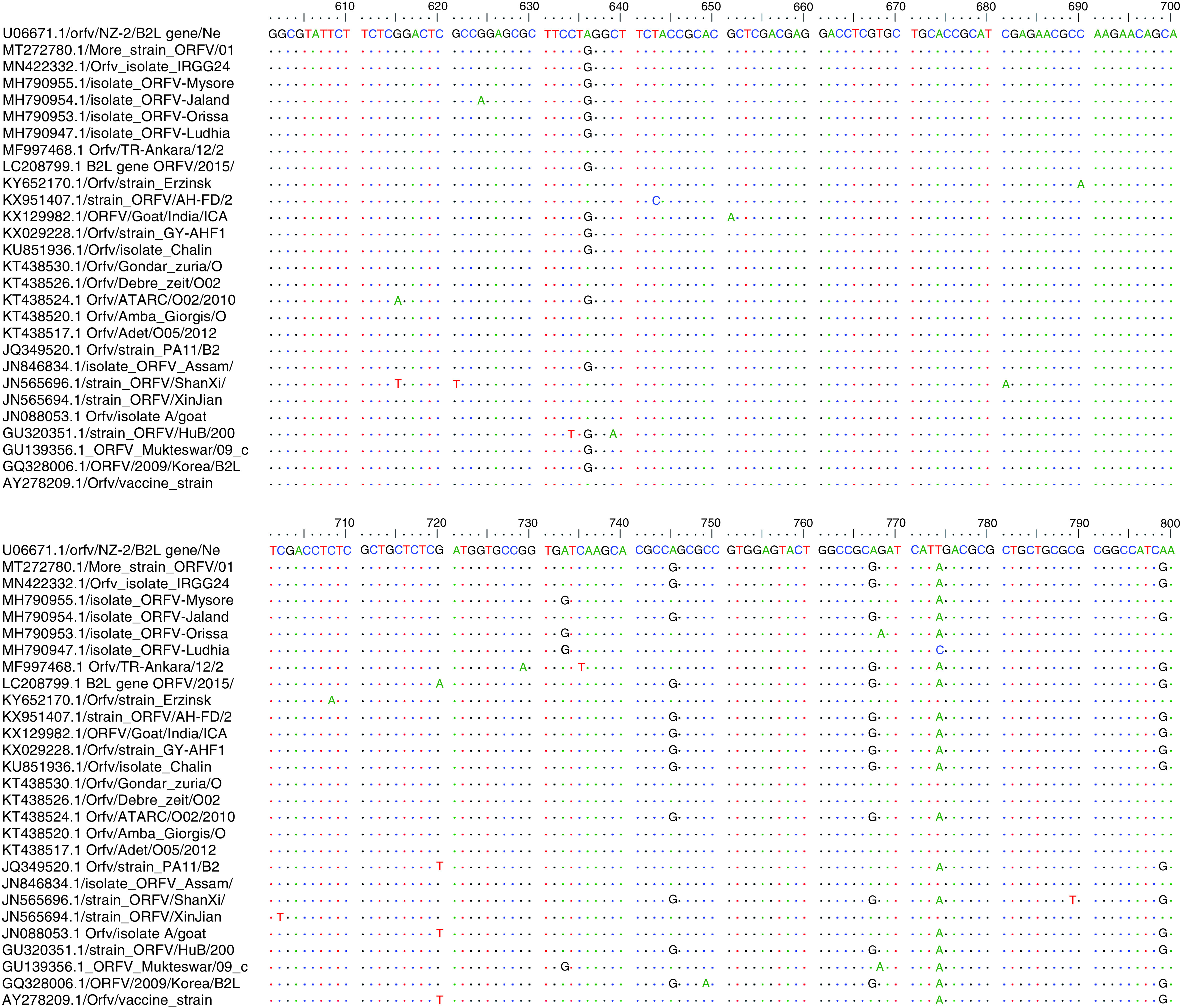

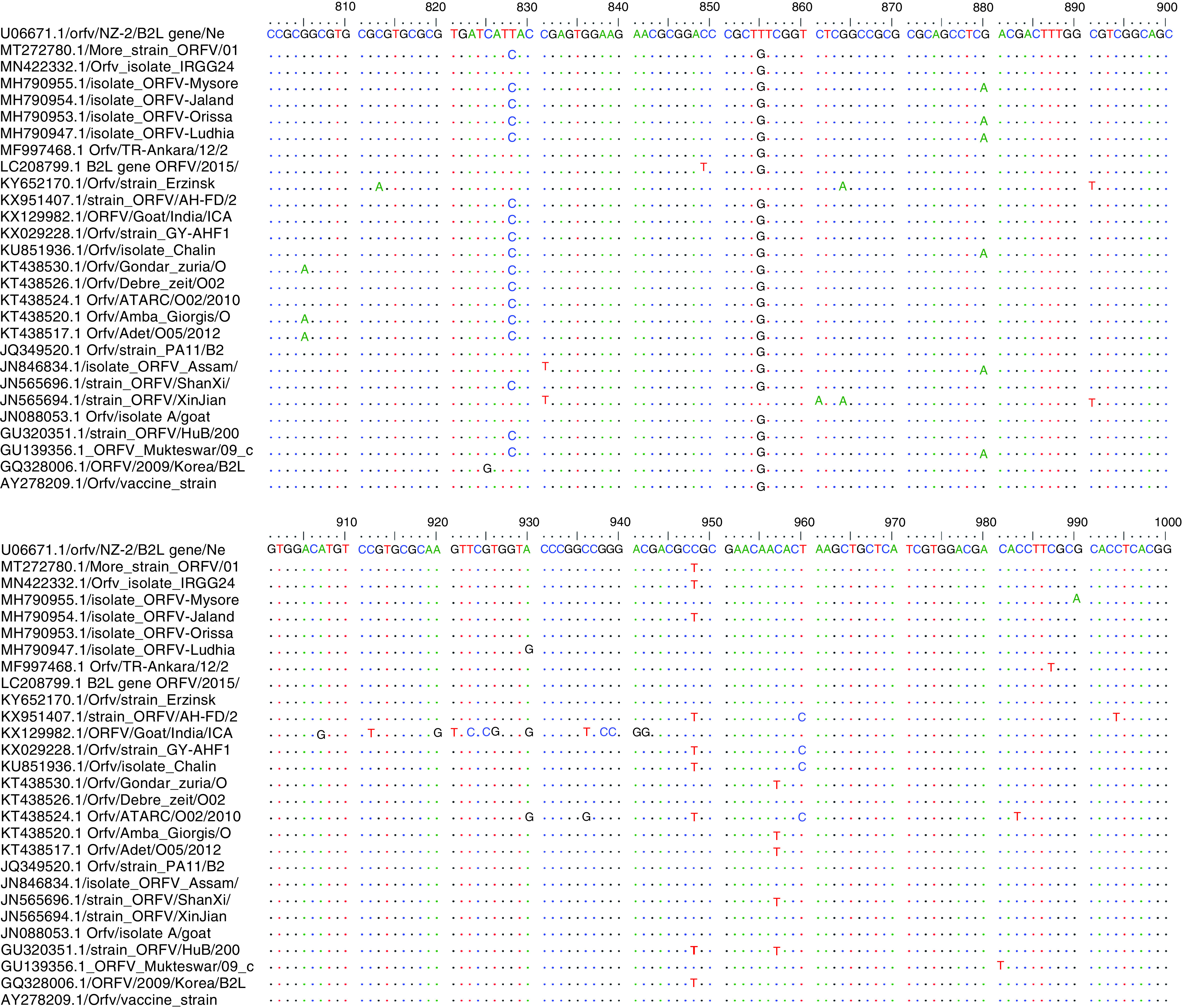

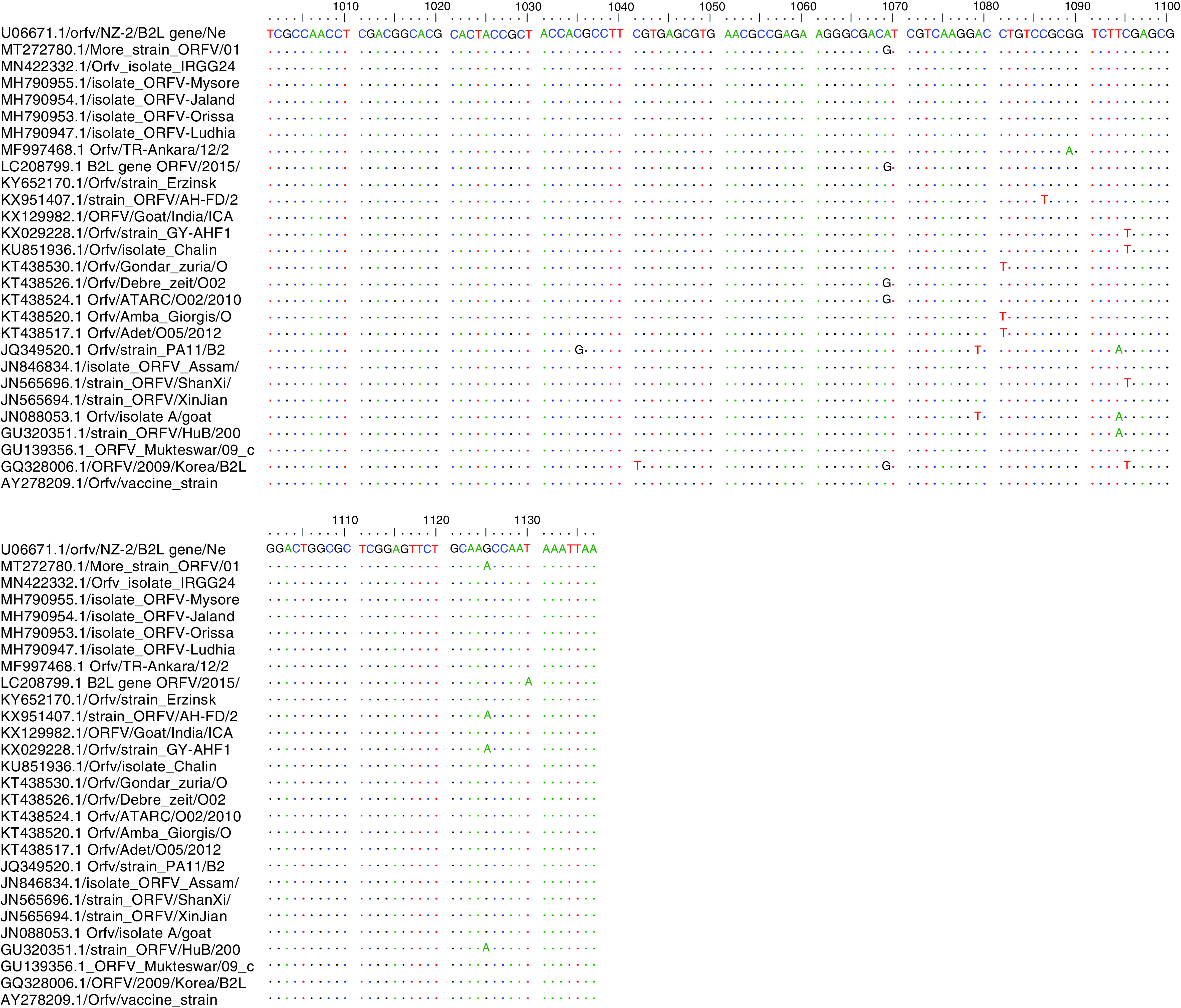
Nucleotide sequence comparison between More strain and reference sequences downloaded from the GeneBank: NCBI. The strain ORFV/NZ-2 from New Zealand (accession number U06671.1) was used as the guide sequence. Areas of similarity with guide sequence were represented as dots (….), while areas of differences were represented by a letter denoting the nucleotide.

**Figure 4.**
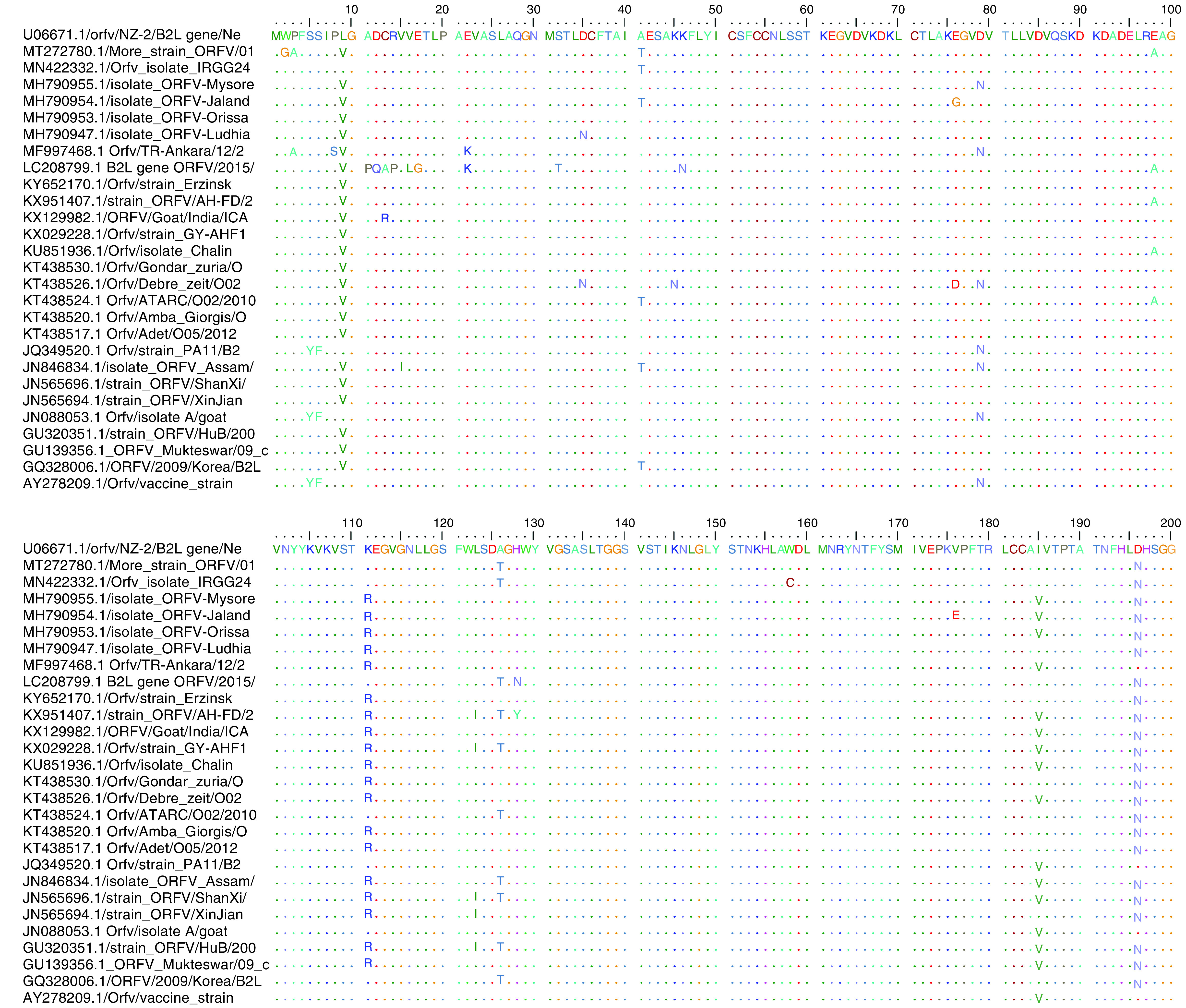

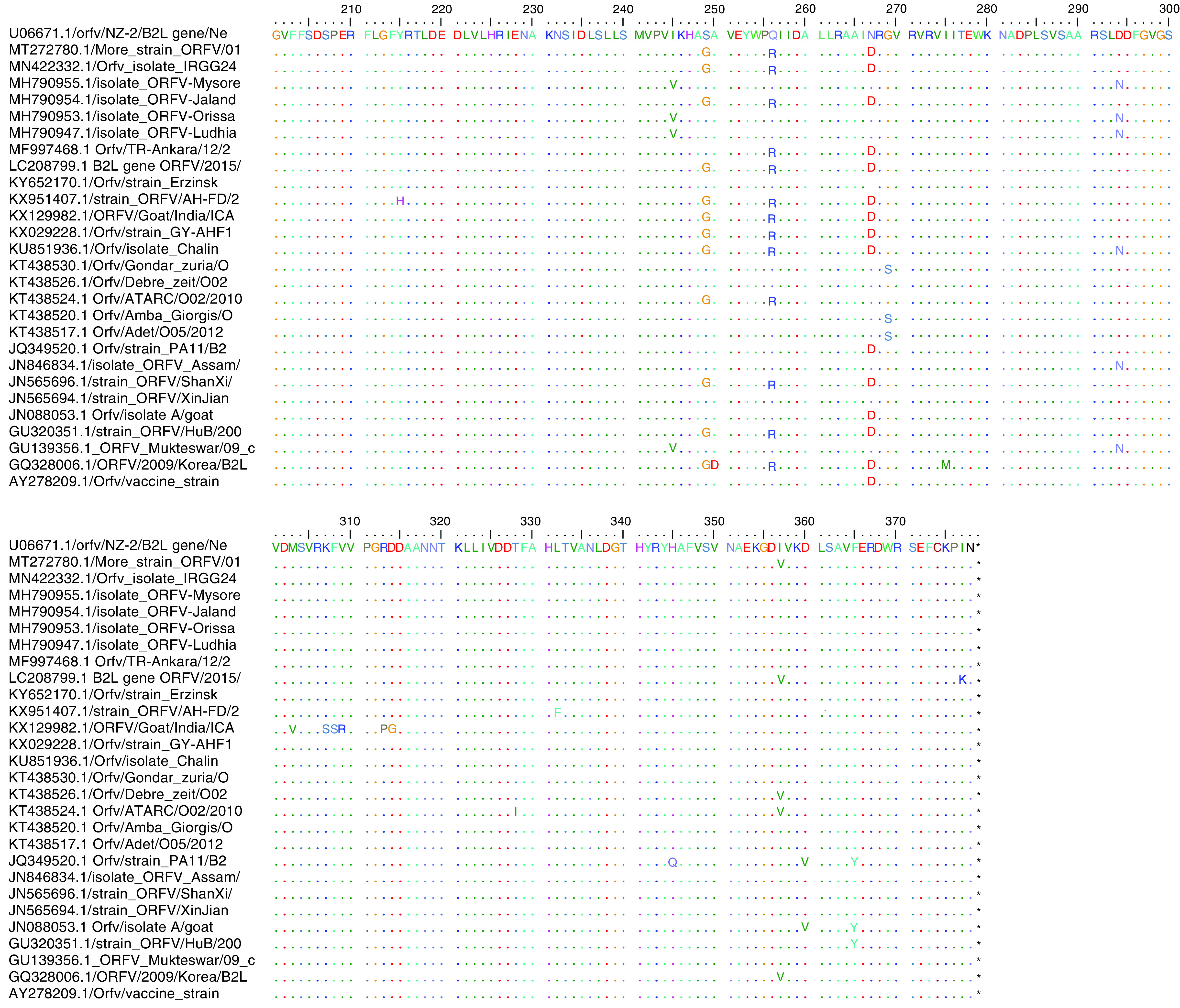
Amino acid sequence comparison between More_strain and reference sequences downloaded from the GeneBank: NCBI. The strain ORFV/NZ-2 from New Zealand (accession number U06671.1) was used as the guide sequence. Areas of similarity with guide sequence were represented as dots (….), while areas of differences were represented by a letter denoting the amino acid.

When the obtained PCR products were sent for sequencing, only sample 2 from the RSG (product in well 4) passed the quality assurance (QC) test necessary for a successful sequencing services possibly because of the high intensity of the band obtained by PCR amplification (Figure 2) and some inhibitors and impurities that may degrade the PCR product from sample 1. Sample 2 was, therefore, the only product sequenced in both directions using the *B2L* forward and reverse primers. Subsequently, the sequence obtained was subjected to BLAST search in the NCBI database and the identity was confirmed as orf virus which was named ‘More_strain’. The sequence was aligned with downloaded orf virus reference sequences at nucleotide (Figure 3) and amino acid levels (Figure 4) to observe for similarities and differences in the nucleotide and amino acid sequences.

Comparison of the obtained sequence with reference sequences from different countries using Tamura–Nei model revealed nucleotide similarities range of 96.7–99.0% among the sequences. Similarly, at the amino acid level, the percentage homologies ranged from 95.7 to 98.9% between the Nigerian orf virus isolate and the reference sequences. At the amino acid level, several mutations were observed in the More strain compared with the New Zealand NZ-2 strain used as a reference guide. There was a W2G at position 2 that was unique to the isolate studied, P3A present only in Ankara strain (MF997468), L9V present in almost all the other reference sequences, A41T which More strain share with five other reference sequences (MN422332; MH790954; KT438524; JN846834 and CQ328006), E98A found in four other reference sequences (LC208799; KX951407; KU851936 and KT438524) and A126T found also in nine other reference sequences (MN422332; LC208799; KX951407; KX029228; KT438524; JN846838; JN565696; GU320351 and GQ328006). Other substitution mutations found were at amino acid positions D196N present in More_strain and all other reference sequences except few (MF997468; JQ349520; JN088053 and the vaccine strain AY278209), S249G, Q256R, N267D and finally I352V (Figure 4).

**Figure 5.**
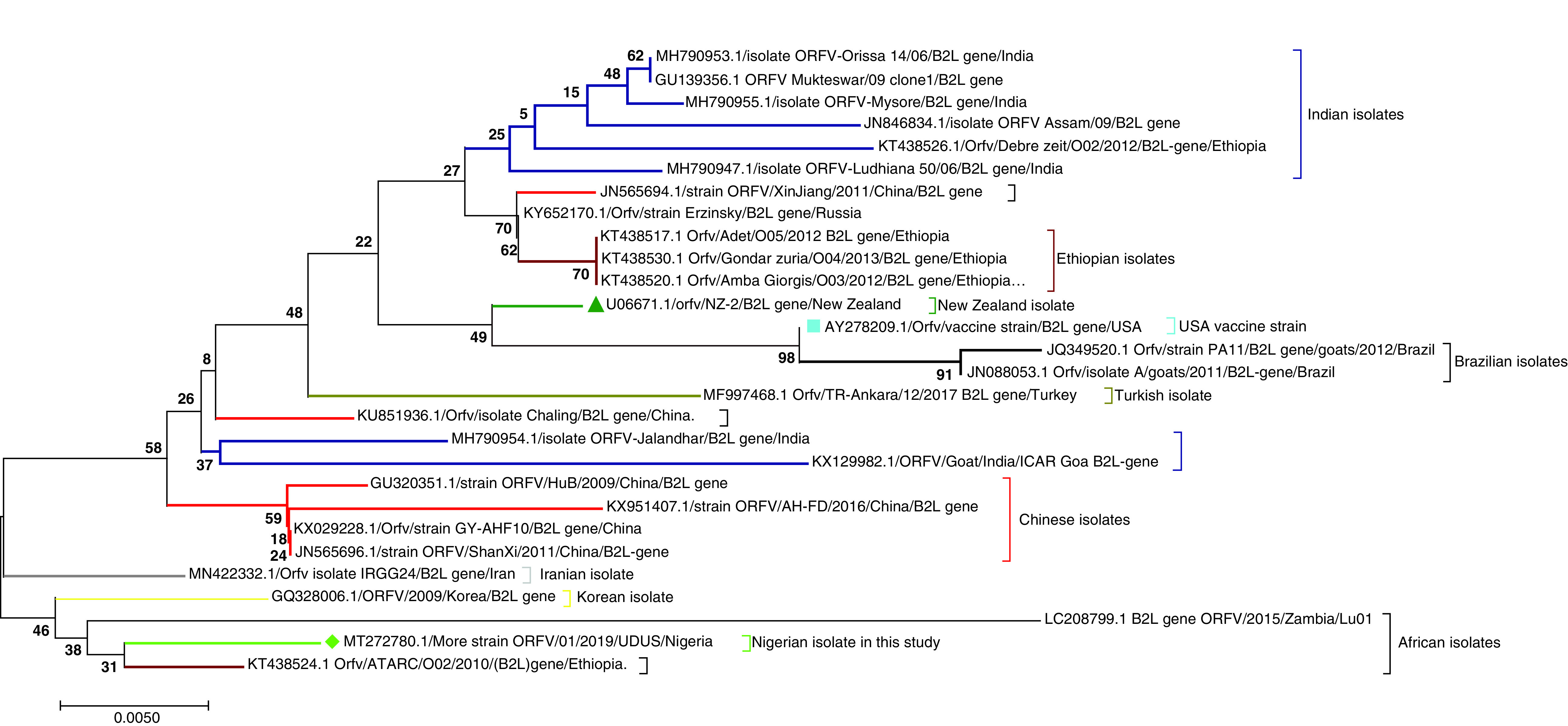
Evolutionary relationships of taxa. The figure showed the phylogenetic relationship between the Nigerian orf virus isolate ‘More_strain’ and other reference sequences from different parts of the world. The Nigerian More_strain clustered together more closely with Ethiopian and Zambian strains and the Iranian IRGG24 isolate is their common most recent ancestor. The evolutionary history was inferred using the neighbor-joining method with 2000 replicates bootstrap. The evolutionary distances were computed using the Poisson correction method involving 28 amino acid sequences. There were a total of 378 positions in the final dataset. Evolutionary analyses were conducted in MEGA7.

Phylogenetic tree analysis based on the *B2L* gene showed that More_strain was closely related to the Ethiopian isolate (KT438524) which explained the high degree of nucleotide and amino acid similarities of 99.0 and 98.9% respectively that exist between them. They form a distinct cluster together with the isolates from Iran (MN422332), Korea (GQ328006) and Zambia (LC208799) (Figure 5). The Iranian strain was the most recent common ancestor to the Korean, Zambian, Ethiopian and More_strains. Other isolates from diverse geographical locations (Asia, Africa, Europe, America and New Zealand) formed separate clusters and subclusters indicating a wide range of genetic diversity that exist in orf viruses.

## Discussion

Orf or contagious ecthyma is endemic in Nigeria and causes huge economic losses in the national livestock industry [4] just as are other bacterial diseases such as brucellosis [32], yersiniosis [33] and parasitic infections such as hydatidosis [34] and gastrointestinal nematodes [35,36]. Effective control of the disease, therefore, demands early detection and identification of the causative agent. In Nigeria, outbreaks of CE are largely documented based on clinical signs which unfortunately may be confused with infection resulting from other viral disease agents such as sheep poxvirus, foot and mouth disease virus, bovine herpes virus type-2 and bluetongue virus. Thus, reliable estimate of the true prevalence of the disease in the country is currently lacking. In recent times, different techniques such as physical examination for clinical signs and PCR have been used to confirm contagious ecthyma outbreaks in Nigeria [1,2,4]. Furthermore, previous study conducted in 2018 involving three flocks of goats in northern Nigeria reported a mortality rate of 100% [4] in two out of the three flocks investigated, which was higher than usually observed in CE outbreaks. This necessitated the need to molecularly characterize the orf virus circulating within the northern region of Nigeria in order to appreciate the relationship between Nigerian isolates and other ones prevalent in other parts of the world. Moreover, to the best of our knowledge, detailed molecular epidemiological studies of CE has not been carried out in northern Nigeria. Consequently, the present study was designed to isolate and genetically characterize Orf virus obtained from an outbreak involving a goat farm in Sokoto metropolis, northwestern Nigeria.

Between May and September 2019, a number of suspected CE outbreaks have occurred in Sokoto State, Nigeria. One of those outbreaks involved a goat farm located in More village within Sokoto metropolis. Scabs were obtained from the two affected animals and prepared for inoculation into CEE via the CAM route for virus isolation. Expectedly, the CAM developed characteristic pock lesions typical of orf viruses, signifying virus replication. Although, this method of virus isolation is simple and relatively rapid for orf diagnosis, to the best of our knowledge, this is the first report of orf virus isolation using specific antibody free CEE in Nigeria.

The full length *B2L* gene sequence obtained when blasted confirmed the identity of the virus isolate as orf virus using the NCBI BLASTN tool. The virus when aligned with deposited orf virus sequences downloaded from NCBI database showed similarities with the reference sequences at both nucleotide and amino acid levels. A unique amino acid change observed only in the strain under study was the W2G seen at amino acid position 2. Another mutation observed was the P3A change at position 3 that was seen only in the Ankara strain (MF997468) from Turkey. However, the significance of these two substitution mutations as they affect the virulence and pathogenicity of the orf virus isolate under study need to be investigated. This is necessary in order to determine the extent of genetic diversity of the orf virus isolates circulating in Sokoto state and its environs since no vaccination against CE are currently practiced in the country. Moreover, the number of samples analyzed in the current study is not enough to determine the significance of the mutations observed, this can be achieved by a larger study covering the whole state.

Phylogenetic tree analysis based on the *B2L* gene showed four major branches on the tree, with the isolate under study (More_strain, MT272780.1) forming a distinct cluster with Korean (GQ328006.1), Zambian (LC208799.1) and Ethiopian (KT438524.1) isolates, suggesting a common ancestor. Indeed, the Nigerian isolate and Ethiopian (KT438524.1) isolate are so phylogenetically related that they are believed to share a common most recent ancestor during evolution. Expectedly, a high degree of nucleotide and amino acid similarities of 99.0 and 98.9% respectively was demonstrated between the two isolates. Another branch on the tree had four major sub-branches where Chinese isolates (red line) diverged from the others, while the New Zealand (U06671), USA vaccine (AY278209) and two Brazilian strains (JQ349520 and JN088053) clustered together (green line) in a separate branch. Isolates from India (blue line) clustered together with one Ethiopian strain in a separate sub-branch. Finally, three Ethiopian isolates clustered with Erzinsky strain (KY652170) from Russia and XinJiang strain (JN565694) from China in a separate sub-branch. This analysis indicated that the Nigerian isolate investigated is genetically different with other ORFV isolated from different geographic regions of the world but closely related with other orf viruses circulating in Ethiopia, Zambia and Korea. The nucleotide and amino acid sequence analyses conducted in this study further confirmed usefulness of the *B2L* gene for genetic and phylogenetic analysis due to its conserved nature and the percentage similarity at both nucleotide and amino acid being in agreement with previous studies [3,37–39]. However, there is sufficient mutation to allow for genetic differentiation between the Nigerian isolate and the reference sequences. The presence of some certain mutations at position 196 (D-N), 256 (Q-R) and 267 (N-D) observed in this study was previously reported by Olivero *et al*. in Uruguayan orf viruses [3] indicating their genetic relatedness of the Nigerian isolate. The absence of the sheep specific conserved residue S at position 249 [37] in the isolate under study may indicate that the G residue found in that same amino acid position may be regarded as goat specific residue to be found in isolates that have adapted to goats. However, this assertion needs to be confirmed by further studies using several orf virus *B2L* gene sequences isolated from goats. The presence and significance of the unique mutation at position W2G as well as P3A present in only the Turkish Ankara strain (MF997468) among the reference sequences examined needs to be further investigated by both *in vivo* and *in vitro* studies in relation to virulence and pathogenicity This study provided the first insight on the genetic diversity of the orf viruses circulating within the Nigerian goat population using complete *B2L* gene nucleotide and amino acid sequence analyses as well as phylogenetic approach.

## Conclusion

In summary, the authors have examined the genetic characteristics of orf virus causing disease outbreaks in Sokoto metropolis, Nigeria and provided molecular signatures of the virus in the study area. The authors showed that the virus under study shared a close genetic relationship with an isolate from Ethiopia and harbors an amino acid change (W2G) that is unique to it and another mutation at P3A that is present in only in the Ankara strain (MF997468). We further highlighted the possibility of the G249 residue as being goat specific conserved residue in the same way as S249 is sheep specific conserved residue. This information is crucial to understand the molecular epidemiology of orf virus circulating in Nigeria, which is necessary for the design of effective CE vaccines which are currently lacking in the country.

## Future perspective

There is the need to undertake a state-wide isolation and characterization study to fully understand the molecular epidemiology of the orf virus isolates circulating within the goat and sheep population in Nigeria. Whole genome sequencing can be done to add to the existing orf virus complete genome sequences since, currently, very few are in the public database. The significance of the W2G and P3A mutations with respect to orf virus virulence modulation and pathogenicity needs to be investigated.

**Summary points**

- Scab lesions from goats suspected to be infected with orf virus based on clinical signs were obtained.
- Genomic DNA was extracted and amplified by PCR.
- The PCR products were analyzed by gel electrophoresis and sent for sequencing.
- The sequencing result was analyzed and aligned using ClustalW to determine its similarity with reference sequences downloaded from GeneBank.
- Deduced amino acid was determined and any change between the isolate and the reference sequences were noted.
- Phylogenetic tree was constructed to determine the taxonomic relationship between taxa.
- One unique amino acid change (W2G) and another (P3A) present only in a Turkish isolate were observed in the isolate under study.
- Phylogenetically, the isolate is more closely related to the Ethiopian isolate (KT438524) with which it clusters in the same clade different from other reference sequences.
- This is the first report on the molecular characterization of orf virus using full length *B2L* gene sequences.

## References

[1] Adedeji AJ, Maurice NA, Wungak YS Diagnosis of orf in west African dwarf goats in Uyo, Akwa Ibom state, Nigeria. African J. Infect. Dis. 11(2), 90–94 (2017). 10.21010/ajid.v11i2.12 PMC5476818 28670645

[2] Adedeji AJ, Gamawa AA, Chima NC First report of camel contagious ecthyma in Nigeria. Open Vet. J. 8(2), 208 (2018). 30425954 10.4314/ovj.v8i2.16 PMC6202667

[3] Olivero N, Reolon E, Arbiza J, Berois M. Genetic diversity of orf virus isolated from sheep in Uruguay. Arch. Virol. (2018). 10.1007/s00705-018-3717-x 29368063

[4] Adedeji AJ, Adole JA, Chima NC Contagious ecthyma in three flocks of goats in Jos-south LGA, Plateau State, Nigeria. Sokoto J. Vet. Sci. 16(1), 107 (2018).

[5] Yang H, Meng Q, Qiao J Short communication detection of genetic variations in orf virus isolates epidemic in Xinjiang China. J. Basic Microbiol. 54, 1–6 (2014). 24633847 10.1002/jobm.201300911

[6] Wang G, Wang Y, Kong J, Li Y, Wu J, Chen Y. Comparison of the sensitivity of three cell cultures to ORFV. BMC Vet. Res. 15(13), 4–11 (2019). 30616567 10.1186/s12917-018-1760-1 PMC6322270

[7] Bala AJ, BalaKrishnan NK, Firdaus FJ Identification of strain diversity and phylogenetic analysis based on two major essential proteins of orf viruses isolated from several clinical cases reported in Malaysia. Infect. Genet. Evol. 77 (September 2019), 104076 (2020). 31678648 10.1016/j.meegid.2019.104076

[8] Tedla M, Berhan N, Molla W, Temesgen W, Alemu S. Molecular identification and investigations of contagious ecthyma (orf virus) in small ruminants, North west Ethiopia. BMC Vet. Res. 14(1), 1–8 (2018). 29334948 10.1186/s12917-018-1339-x PMC5769459

[9] Mazur C, Machado RD. Detection of contagious pustular dermatitis virus of goats in a severe outbreak. Vet. Rec. 125(16), 419 LP–420 (1989). 2588444 10.1136/vr.125.16.419

[10] Bayindir Y, Bayraktar M, Karadag N Investigation and analysis of a human orf outbreak among people living on the same farm. New Microbiol. 34(1), 37–43 (2011). 21344145

[11] Haddock ES, Cheng EC, Bradley SJ Extensive orf infection in a toddler with associated id reaction. Pediatr. Dermatol. 34(6), e337–340 (2017). 28940500 10.1111/pde.13259 PMC6423519

[12] Nagarajan G, Pourouchottamane R, Reddy GBM Molecular characterization of orf virus isolates from Kodai hills, Tamil Nadu, India. Vet. World 12(7), 1022–1027 (2019). 31528027 10.14202/vetworld.2019.1022-1027 PMC6702573

[13] Chan KW, Lin JW, Lee SH Identification and phylogenetic analysis of orf virus from goats in Taiwan. Virus Genes 35(3), 705–712 (2007). 17682935 10.1007/s11262-007-0144-6

[14] Riccardo W, Clive CK, Robert W. High C + G Content in Parapoxvirus I) N A. J. Gen. Virol. 43, 231–234 (1979). 225418 10.1099/0022-1317-43-1-231

[15] Fleming SB, Wise LM, Mercer AA. Molecular genetic analysis of orf virus: a poxvirus that has adapted to skin. Viruses 7, 1505–1539 (2015). 25807056 10.3390/v7031505 PMC4379583

[16] Bala JA, Balakrishnan KN, Abdullah AA Dermatopathology of orf virus (Malaysian Isolates) in mice experimentally inoculated at different sites with and without dexamethasone administration. J. Pathog. 2018, 1–12 (2018). 10.1155/2018/9207576 PMC6093002 30155311

[17] Gelaye E, Achenbach JE, Jenberie S, Ayelet G, Belay A. Molecular characterization of orf virus from sheep and goats in Ethiopia, 2008–2013. Virol. J. 2008–13 (2016). 10.1186/s12985-016-0489-3 PMC4770539 26923232

[18] Hosamani M, Scagliarini A, Bhanuprakash V, McInnes CJ, Singh RK. Orf: an update on current research and future perspectives. Expert. Rev. Anti. Infect. Ther. 7(7), 879–893 (2009). 19735227 10.1586/eri.09.64

[19] Zhang K, Lu Z, Shang Y Diagnosis and phylogenetic analysis of orf virus from goats in China: acase report. Virol. J. 7(78), 1–5 (2010). 20416112 10.1186/1743-422X-7-78 PMC2877020

[20] Zhang K, Liu Y, Kong H. Comparison and phylogenetic analysis based on the B2L gene of orf virus from goats and sheep in China during 2009-2011. Arch. Virol. 159(6), 1475–1479 (2014). 24343266 10.1007/s00705-013-1946-6 PMC4042016

[21] Mwanandota JJ, Macharia M, Sallu R, Yongolo M, Holton TA. Phylogenetic analysis of orf virus from goats in Tanzania. Univ. J. Agric. Res. 4(5), 165–169 (2016).

[22] Ferede Y, Habtamu A, Gebresellasie S. Confirmatory diagnosis of contagious ecthyma (orf) by polymerase chain reaction at Adet Sheep Research Sub-Center, Ethiopia: a case report. J. Vet. Med. Anim. Health 6(July), 187–191 (2014).

[23] Mahmoud M, Soliman H. Molecular and virological studies on contagious pustular dermatitis isolates from Egyptian sheep and goats. Res. Vet. Sci. 89(2), 290–294 (2010). 20304450 10.1016/j.rvsc.2010.02.019

[24] Zeedan GSG, Abdalhamed AM, Ghoneim NH, Ghazy AA. Isolation and molecular diagnosis of orf virus from small ruminants and human in Egypt. J. Antivir. Antiretrov. 7(1), 2–9 (2015).

[25] Maganga GD, Relmy A, Bakkali-kassimi L Molecular characterization of orf virus in goats in Gabon, Central Africa. Virol. J. 1–5 (2016). 27178401 10.1186/s12985-016-0535-1 PMC4866431

[26] Khalafalla AI, El-sabagh IM, Al-busada KA, Al-mubarak AI, Ali YH. Phylogenetic analysis of eight sudanese camel contagious ecthyma viruses based on B2L gene sequence. Virol. J. 1–9 (2015). 26260127 10.1186/s12985-015-0348-7 PMC4578853

[27] Ibrahim A, Eisa A, Zackaria H, Ishag A. Field investigation and phylogenetic characterization of orf virus (ORFV) circulating in small ruminants and pseudocowpoxvirus (PCPV) in dromedary camels of eastern Sudan. Heliyon 6(November 2019), e03595 (2020). 32258461 10.1016/j.heliyon.2020.e03595 PMC7096746

[28] Lawal N, Hair-Bejo M, Arshad SS, Omar AR, Ideris A. Adaptation and molecular characterization of two malaysian very virulent infectious bursal disease virus isolates adapted in BGM-70 cell Line. Adv. Virol. 2017 1–19 (2017). 10.1155/2017/8359047 PMC5694579 29230245

[29] Hosamani M, Bhanuprakash V, Scagliarini A, Singh RK. Comparative sequence analysis of major envelope protein gene (B2L) of Indian Orf viruses isolated from sheep and goats. Vet. Microbiol. 116(4), 317–324 (2006). 16777357 10.1016/j.vetmic.2006.04.028

[30] Lawal N, Hair-Bejo M, Arshad SS, Omar AR, Ideris A. Propagation and molecular characterization of bioreactor adapted very virulent infectious bursal disease virus isolates of Malaysia. J. Pathog. 2018, 11 (Article ID 1068758). 10.1155/2018/1068758 PMC6139196 30245887

[31] Kumar S, Stecher G, Tamura K. MEGA7: molecular evolutionary genetics analysis version 7.0 for bigger datasets. Mol. Biol. Evol. 33(7), 1870–1874 (2016). 27004904 10.1093/molbev/msw054 PMC8210823

[32] Lawal N, Egwu GO, Tambuwal FM Prevalence of *brucella abortus* antibodies in bovine serum from gusau modern abattoir, Zamfara state, Nigeria. Sci. J. Microbiol. 1(4), 91–96 (2012).

[33] Jibrin MS, Faleke OO, Salihu MD Prevalence of *Yersinia enterocolitica* and *Yersinia pseudotuberculosis* in pigs in Zuru local government area, Kebbi state. Sci. J. Vet. Adv. 2(12), 189–196 (2013).

[34] Saulawa MA, Magaji AA, Faleke OO Serodiagnosis of hydatidosis in sheep slaughtered at Sokoto abattoir, Sokoto state, Nigeria. Sokoto J. Vet. Sci. 9(2), 20–23 (2011).

[35] Saulawa MA, Magaji AA, Faleke OO Prevalence of *Cysticercus tenuicollis* cyst in sheep slaughtered at Sokoto abattoir, Sokoto state, Nigeria. Sokoto J. Vet. Sci. 9(2), 24–27 (2011).

[36] Mahmuda A, Yakubu Y, Raji AA Prevalence of gastrointestinal round worms in calves in Sokoto, northwestern Nigeria. Sci. J. Zoology 1(2), 26–30 (2012).

[37] Abrahão JS, Borges I, Mazur C Looking back: a genetic retrospective study of Brazilian Orf virus isolates. Vet. Rec. 171(19), 476 (2012). 23065256 10.1136/vr.100634

[38] Kumar N, Wadhwa A, Chaubey KK Isolation and phylogenetic analysis of an orf virus from sheep in Makhdoom, India. Virus Genes 48, 312–319 (2014). 24347045 10.1007/s11262-013-1025-9

[39] Zhao K, Song D, He W Identification and phylogenetic analysis of an orf virus isolated from an outbreak in sheep in the Jilin province of China. Vet. Micro. 142, 408–415 (2010). 10.1016/j.vetmic.2009.10.006 19948384

